# Supplementary material for: Small target detection algorithm based on the fusion attention mechanism and multi-layer convolution
Source: PLoS One. 2025 Jul 31;20(7):e0328003. doi: 10.1371/journal.pone.0328003 (PMC12312941; doi:10.1371/journal.pone.0328003)
Supplement: Attachment — Submitted filename: Comments-plos one.docx [file pone.0328003.s001.docx]

The manuscript presents a novel algorithm, MGAC-YOLO, for small target detection in UAV applications, demonstrating improvements over existing models. The article has a well-structured format, and the language and article structure are generally good. However, there are some minor problems to be addressed, below are detailed recommendations for improvement.

Minor comments

1. The description of the GACAttention module's integration strategy is not clearly, and experimental hyperparameters (e.g., learning rate decay strategy) is inadequate. More technical details are needed to improve the study's reproducibility.
2. It is recommended to add a discussion on the model's computational efficiency or real - time limitations in the conclusion section. This will enhance the study's completeness and practicality.
3. Definitions and formatting in formulas (1) and (2) are non-standard. eg: "SiLu" and "SiLU" are used inconsistently.
4. The figures are numbered in confusion, such as: the subfigures of Figure 10 are not clearly referenced in the text.
5. Some references are missing volume numbers or page numbers (e.g., Reference [7], [11]). Please conduct a thorough check of the reference format.
6. The manuscript inconsistently uses "YOLOv8" and "YOLOV8", requiring standardization to "YOLOv8" for professional consistency.
